# Myasthenia gravis with anti-muscle-specific tyrosine kinase antibodies during therapy for multiple myeloma: a case report

**DOI:** 10.1186/s12883-020-01813-1

**Published:** 2020-06-12

**Authors:** Shoko Sakano, Hirofumi Matsuyama, Hidehiro Ishikawa, Akihiro Shindo, Yuichiro Ii, Keita Matsuura, Minoru Mizutani, Norikazu Kawada, Hidekazu Tomimoto

**Affiliations:** 1 Department of Neurology, Mie University Graduate School of Medicine, 2-174 Edobashi, Tsu, Mie 514-8507 Japan; 2 Department of Hematology, Matsusaka Central General Hospital, 102 Azakomon, Kawaimachi, Matsusaka, Mie 515-0818 Japan; 3 Department of Neurology, Matsusaka Central General Hospital, 102 Azakomon, Kawaimachi, Matsusaka, Mie 515-0818 Japan

**Keywords:** Myasthenia gravis, Anti-muscle-specific tyrosine kinase antibodies, Multiple myeloma, Bortezomib, Case report

## Abstract

**Background:**

The onset of myasthenia (MG) gravis with anti-muscle-specific tyrosine kinase (MuSK) antibodies most commonly peaks in the fourth decade of life, and MG with MuSK antibodies (MuSK-MG) rarely coexists with a malignant tumor. To date, MuSK-MG has not been reported in multiple myeloma (MM).

**Case presentation:**

A 60-year-old male with MM who was receiving treatment with bortezomib and thalidomide presented diplopia, ptosis, and limb weakness. A diagnosis of MM with Bence-Jones proteinuria was established when he was 56 years old, and he received chemotherapy with four courses of bortezomib and dexamethasone. Although he received thalidomide as maintenance therapy, it was discontinued a year before hospital admission because of sensory neuropathy as a side effect. Six months before hospital admission, he developed mild diplopia. One month before admission, his chemotherapy was interrupted because of viral infection and fatigability. Then he developed neck weakness and bilateral ptosis. A diagnosis of MuSK-MG was made based on neurological and serological examinations. According to the previous relevant literature, this is the first report of MuSK-MG in a patient with MM.

**Conclusions:**

In patients with MM, the possibility of co-existing of autoimmune disease, including MuSK-MG, should be considered. This case emphasizes the need to still consider testing for anti-MuSK antibodies in older MM patients where there is clinical suspicion for possible MG despite negative anti-acetylcholine receptor antibodies and lacking classic MuSK MG phenotype at onset.

## Background

The onset of myasthenia gravis with anti-muscle-specific tyrosine kinase antibodies (MuSK-MG) most commonly peaks in the late 30s, and an onset at a later age is unusual [1, 2]. Patients with MuSK-MG often present with bulbar symptoms, and extremity weakness is uncommon [1]. It is extremely rare for MuSK-MG to coexist with thymoma or a malignant tumor. This report describes a 60-year-old male patient who developed MuSK-MG during therapy for multiple myeloma (MM).

## Case presentation

**Fig. 1:**
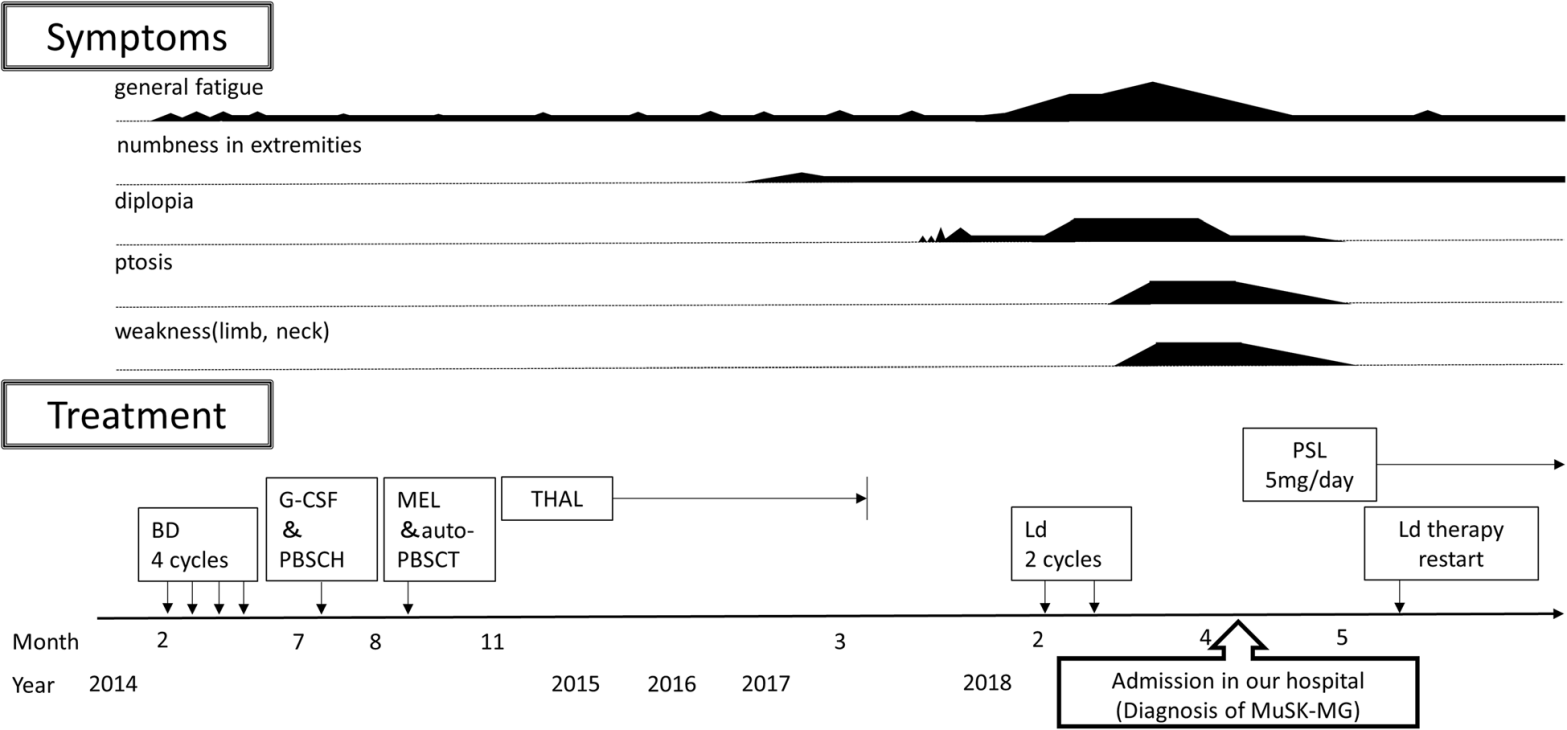
Clinical course. In 2014, an induction therapy consisting of 4 cycles of 21-day bortezomib plus a dexamethasone (BD) regimen (intravenous bortezomib 1.3 mg/m^2^ on days 1, 4, 8, and 11; and oral dexamethasone 20 mg/day on days 1, 2, 4, 5, 9, 11, and 12) was administered. Granulocyte-colony stimulating factor (G-CSF) with peripheral blood stem cell harvesting (PBSCH) and melphalan (MEL) with auto- peripheral blood stem cell transplantation (PBSCT) were performed subsequently. In auto-PBSCT, a total of 200 mg/m^2^ MEL was administered over two consecutive days (100 mg/m^2^/day) as a conditioning prior to auto-PBSCT. Thalidomide (THAL) 100 mg/day was given as maintenance therapy until it was stopped due to side effects. In 2018, a maintenance therapy consisting of 28-day lenalidomide plus a low-dose dexamethasone (Ld) regimen (lenalidomide 10 mg/day on days 1–21 plus dexamethasone 20 mg/d; days 1, 8, 15, and 22) was initiated. After diagnosis of myasthenia gravis with anti-muscle-specific tyrosine kinase antibodies (MuSK-MG), oral prednisolone (PSL) 5 mg daily was started

A 60-year-old man came to our hospital with diplopia, ptosis, and fatigue. A diagnosis of MM with Bence-Jones proteinuria was established when he was 56. His bone marrow biopsy revealed hypercellular tissue with > 70% of CD138 positive cells. The biopsy was negative for CD20 and CD3, and was consistent with plasma cell myeloma. Blot clonality was not observed on immunoelectrophoresis. He had received chemotherapy with bortezomib and dexamethasone, followed by other drugs and agents (Fig. 1). Although he was treated with thalidomide as maintenance therapy, that was discontinued 1 year before hospital admission because of sensory neuropathy side effects. Six months prior to hospital admission, he developed transient diplopia which he observed sporadically while performing desk work. His investigations at a neurology outpatient clinic did not detect anti-acetylcholine receptor (AChR) antibodies on radioimmunoassay and thyroid function was normal. Brain magnetic resonance (MR) imaging showed no causative abnormalities including extraocular muscles. A severe stenosis of the right middle cerebral artery was serendipitously found on the head MR angiography, and he was treated surgically, but the diplopia did not improve. Two months before hospital admission, he received two cycles of lenalidomide and dexamethasone for MM. One month prior to admission, he appeared to have developed viral upper tract infection, which was followed by fatigability and necessitated stoppage of his chemotherapy. In the last month prior to admission, he gradually developed mild neck weakness, persistent diplopia, and bilateral ptosis. At admission, neurological examination revealed bilateral ptosis, diplopia on lateral gaze, bilateral limitation in upward and lateral gaze, mild limb weakness, and dysesthesia. Deep tendon reflexes were within normal limits, and no autonomic abnormalities were noted. Functional respiratory tests showed values for vital capacity and forced expiratory volume in 1 sec within normal limits. Swallowing was also normal. Blood testing revealed a serum anti-MuSK antibody level of 21.6 nmol/L (normal, < 0.05 nmol/L). The amplitude of the compound muscle action potential showed > 10% decrement on repetitive nerve stimulation (RNS) for the right nasalis muscle, and the edrophonium test was positive. Computed tomography revealed no thymoma. The patient was diagnosed with MG that was categorized according to the Myasthenia Gravis Foundation of America (MGFA) criteria [3] as Class IIa. His symptoms of general fatigue, diplopia, ptosis and weakness gradually stabilized with the administration of prednisolone (5 mg daily). He left the hospital 27 days after admission with a postintervention status classified as “minimal manifestations” according to the MGFA criteria [3]. The clinical course is presented in Fig. 1.

## Discussion and conclusions

To the best of our knowledge, this is the first report of an MM patient who developed serologically-proven MuSK-MG. This case highlights two important issues. First, MuSK-MG occurred in this case during MM treatment, demonstrating that the symptoms of MuSK-MG might be masked by chemotherapy for MM. Second, onset of ocular complaints in the 60s and limb weakness without bulbar symptoms are atypical presentations of MuSK-MG.

Several cases of MG accompanied by MM or plasmacytoma have been reported in the literature [4–6]. Although the patient reported by Ahmed et al. [5] was, in contrast to our patient, diagnosed with mediastinal plasmacytoma, the primary ocular manifestation was comparable in these cases. In the aforementioned studies, anti-MuSK antibody testing was not performed, and therefore, it remains uncertain whether these patients had MuSK-MG [4–6]. Reportedly, chronic antigenic stimulation of B lymphocytes present in autoimmune disorders might eventually lead to the development of MM [7]. Although the pathophysiological mechanism in the patient described in this case report remains unclear, we hypothesize that an autoimmune condition had already existed before MG manifested clinically. It is possible that his MG symptoms were masked by the treatments of bortezomib and dexamethasone followed by thalidomide [8–11].. Although he received lenalidomide and dexamethasone for MM after the onset of MG, lenalidomide may have been less effective in MG compared to bortezomib [10] and a steroid-induced exacerbation may have aggravated his condition [12]. An alternative hypothesis is that in the present case, B-cell immune dysregulation due to MM might be associated with MG development [13]. In MM patients, abnormal plasma cells produce dysfunctional immunoglobulins, and it is conceivable that those plasma cells produce anti-MuSK antibodies. In our patient, the MM-related findings were stable when he experienced MG symptoms, making the former hypothesis more likely. Since there is an increased prevalence of autoimmune conditions in patients with MM and vice versa [7], it is important to investigate for autoimmune diseases including MuSK-MG in patients with MM.

More than 40% of patients with MuSK-MG present with bulbar weakness as their first symptom. Further, limb weakness is uncommon and ocular muscles are often unaffected [1, 14]. MM can lead to ocular manifestations [15–18], and both progression and treatment of MM can cause weakness and fatigue. Therefore, fatigue and ocular symptoms alone do not automatically imply MG as a possible diagnosis. A previous study reported a 100% incidence of bulbar muscle weakness in MuSK-MG [19]. Our patient presented with limb weakness in the absence of bulbar symptoms, which is atypical for MuSK-MG. Disease onset is usually in the fourth decade of life [20]. The typical disease presentation is as acute illness, although some patients have mild to moderate disease over months before progression to severe MG with persistent bulbar weakness despite immunosuppressive therapy [20]. Our patient’s age was 60 when he noticed ocular symptoms as a part of his disease, which then progressed to include limb weakness. These symptoms were perhaps exacerbated by an infection, since he demonstrated partial improvement as the infection subsided, prior to hospitalization. He required only a small dose of prednisolone (5 mg) to maintain with “minimal manifestations”. The pathogenicity of anti-MuSK antibodies may be different in the presence of MM. There are reports of MuSK-MG being diagnosed later in life, beyond the age of 50 [19, 20]. If clinical syndrome suggests possible neuromuscular junction disorder, comprehensive testing should include anti-MuSK antibodies in addition to RNS and AChR antibodies.

In conclusion, this case emphasizes the need to still consider testing for anti-MuSK antibodies in older MM patients where there is clinical suspicion for possible MG despite negative AChR antibodies and lacking classic MuSK MG phenotype.

## Data Availability

The datasets generated during the current study are available from the corresponding author on reasonable request.

## Abbreviations

MG: Myasthenia gravis
MuSK: Muscle-specific tyrosine kinase (MuSK)
MuSK-MG: Myasthenia gravis with anti-muscle-specific tyrosine kinase antibodies
AChR: Anti-acetylcholine receptor
MR: Magnetic resonance
MGFA: Myasthenia Gravis Foundation of America
